# Matrix stiffness-mediated effects on stemness characteristics occurring in HCC cells

**DOI:** 10.18632/oncotarget.8515

**Published:** 2016-03-31

**Authors:** Yang You, Qiongdan Zheng, Yinying Dong, Xiaoying Xie, Yaohui Wang, Sifan Wu, Lan Zhang, Yingcong Wang, Tongchun Xue, Zhiming Wang, Rongxin Chen, Yanhong Wang, Jiefeng Cui, Zhenggang Ren

**Affiliations:** ^1^ Liver Cancer Institute, Zhongshan Hospital, Fudan University & Key Laboratory of Carcinogenesis and Cancer Invasion, Ministry of Education, Shanghai 200032, PR China; ^2^ Department of Interventional Radiology, Shanghai Cancer Center, Fudan University, Shanghai 200032, PR China; ^3^ Department of Oncology, Zhongshan Hospital Subdivision, Fudan University, Shanghai 200052, PR China

**Keywords:** matrix stiffness, hepatocellular carcinoma, stemness, mTOR, integrin β1

## Abstract

Matrix stiffness as an important physical attribute of extracellular matrix exerts significant impacts on biological behaviors of cancer cells such as growth, proliferation, motility, metabolism and invasion. However, its influence on cancer stemness still remains elusive. Here, we explore whether matrix stiffness-mediated effects on stemness characteristics occur in HCC cells. As the substrate stiffness increased, HCC cells exhibited high proportion of cells with CD133(+)/EpCAM(+), high expression levels of CD133, EpCAM, Nanog and SOX2, greater self-renewing ability and oxaliplatin resistance. Simultaneously, their phosphorylation levels of Akt and mTOR, as well as p-4E-BP and SOX2 expressions were also obviously upregulated. Conversely, knockdown of integrin β1 partially attenuated higher stiffness-mediated stemness characteristics in HCC cells, and reversed the phosphorylation levels of Akt and mTOR, and expressions of p-4E-BP and SOX2, suggesting that integrin β1 may deliver higher stiffness signal into HCC cells and activate mTOR signaling pathway. Additionally, mTOR inhibitor suppressed the mTOR phosphorylation level and expression levels of p-4E-BP and SOX2 in HCC cells grown on higher stiffness substrate, as well as depressed their stemness properties significantly, favoring a regulating role of mTOR signaling pathway in matrix stiffness-mediated effects on stemness. In summary, matrix stiffness may be involved in the process of stemness regulation via activating integrin β1/Akt/mTOR/SOX2 signaling pathway. To the best of our knowledge, this study first reveals a novel regulating pathway to direct the stemness characteristics in HCC cells.

## INTRODUCTION

Hepatocellular carcinoma (HCC) is the fifth most common malignancy in men and ninth most common in women, and also the second leading cause of cancer-related death worldwide [1]. As indicated by clinical data, around 55%–85% of patients with liver cirrhosis develop into HCC, and more than 80% of HCC patients have backgrounds of liver cirrhosis or severe liver fibrosis [2, 3]. Liver matrix stiffness increase is always accompanied by the occurrence and development of chronic liver disease and fibrosis [4, 5]. Currently, liver stiffness measurement as a non-invasive predictor has already been applied in fibrosis diagnosis, risk assessment of HCC development, and recurrence risk stratification after curative resection of HCC [4, 6, 7]. Although a significant correlation between matrix stiffness and HCC has attracted more attention from oncologists in the last two decades, the detailed mechanism by which matrix stiffness-mediated effects on HCC occurrence and progression remains largely unknown. Matrix stiffness is an important physical attribute of solid extracellular matrix (ECM). An increase in matrix stiffness, resulting from abundant matrix protein deposition and crosslinking, not only commonly occurs in the most of solid tumors, but also contributes to cell growth, motility, proliferation, metabolism and even tumor metastasis [8–11]. Some literatures on HCC study indicate that matrix stiffness regulating HCC cell proliferation, drug resistance and angiogenesis might be through focal adhesion kinase (FAK), extracellular-signal-regulated kinase (ERK) /signal transducer and activator of transcription member 3 (STAT3), c-Jun N-terminal kinase (JNK), and phosphatidylinositol-3-kinase (PI3K)/Akt signaling pathways [12–14]. Other studies demonstrate that the mechanical stiffness of ECM remarkably correlates with HCC invasion and metastasis via controlling the expression of integrin β1 [15], and lysyl oxidase 2-mediated tissue stiffness increase promotes intrahepatic metastasis of HCC [16]. In addition, higher matrix stiffness also upregulates osteopontin (OPN) expression in HCC cells by activating integrin β1/GSK3β/β-catenin signaling pathway [17], and forces the expression of vascular endothelial growth factor through integrin β1/AKT/PI3K signaling pathway [14], thereby suggesting that it may participate in the regulation of the expression levels of some invasion/metastasis-associated genes and ultimately influence HCC metastasis. However, little is known on whether higher matrix stiffness mediates effects on stemness characteristics occurring in HCC cells.

Stemness characteristics in cancer cells are associated with a more aggressive disease course and unfavorable clinical outcomes. Cancer stem cell (CSC) hypothesis proposes that a small proportion of cancer cells exhibits stem cell-like characteristics such as self-maintenance, self-renewal and differentiation [18, 19]. Side population (SP) cells isolated from HCC cells, now identified as hepatic CSCs, possess higher proliferation, anti-apoptosis potential and more stemness gene expression compared with non-SP cells. Furthermore, as few as 10^3^ SP cells subcutaneously seeded in NOD/SCID rat could result in tumor formation, thereby revealing that SP cells with low Hoechst staining bear higher tumorigenic ability, the most important functional property of CSCs, to initiate and sustain tumor growth [20, 21]. Except for their higher clonogenicity and tumorigenicity, CSCs also take on other characteristics such as resistance to chemotherapeutics and radiation [18, 22], and are responsible for cancer relapse and metastasis [23]. To date, the existence of CSCs has been validated in some solid tumors including breast [24], brain [25], colorectal [26], lung [27], pancreas [28], liver [29], melanoma [30], prostate cancer [31] and so on. Hoechst 33342 dye is initially used to identify a CSC population in liver cancer cells [32]. Subsequently, other specific surface markers such as cluster of differentiation 133 (CD133), epithelial cell adhesion molecule (EpCAM), CD90, CD44, CD24, CD13, OV6 and their co-expression, as well as aldehyde dehydrogenase (ALDH) activity are applied to isolate or enrich hepatic CSCs from HCC cells [21]. More importantly, hepatic CSCs with a distinct surface marker pattern mentioned above implicate a significance in higher proliferation, tumorigenic potential, high metastasis and poor outcome. Several deregulated signaling pathways such as Wnt/β-catenin, AKT, bone morphogenetic protein 4 (BMP4), Oncostatin M (OSM), B lymphoma Mo-MLV insertion region 1 homolog (Bmi-1), transforming growth factor beta (TGF-beta), interlukin-6 (IL-6)/STAT, and Hedgehog pathways have been well documented to exert a critical role in inducing stemness and in promoting self-renewal, tumorigenicity and chemoresistance of HCC [21, 22, 33–35]. But, little is known about signaling pathway modulating matrix stiffness-mediated stemness characteristic changes in HCC cells. Mechanistic target of rapamycin (mTOR) signaling molecule is required as a critical hub molecule for cell survival and proliferation [36, 37]. This molecule is also involved in the development and progression of many cancers [38]. The activation of mTOR signaling pathway emerges in 40%-50% of HCC patients and indicates their poor prognosis, revealing its pivotal role in HCC progression [39]. Sporadic literatures support the possibility of a linkage between mTOR activity and tumor-initiating cell maintenance or expansion [40–42], and the blockage of Akt/mTOR signaling could diminish cyclin G1-mediated sex determining region Y box 2 (SOX2) induction, and suppress self-renewal, chemoresistance, and tumorigenicity of hepatoma cells [43]. Our previous data also showed that higher phosphorylation levels of AKT and mTOR occur in HCC cells in response to higher matrix stiffness stimulation (data not shown). Therefore, we hypothesized that mTOR signaling pathway may contribute to the regulation of stiffness-mediated HCC stemness.

Here, using an in vitro culture system with tunable stiffness previously reported [17], we investigated the underlying roles of mTOR signaling pathway in matrix stiffness-mediated effects on the stemness characteristics of HCC cells. From the biophysical point of view, this work highlights a novel approach to drive stemness characteristics, and facilitates further studies of targeting matrix stiffness toward the clinical intervention of HCC.

## RESULTS

### Higher matrix stiffness may induce and enhance the stemness characteristics of HCC cells such as the proportion of CD133(+)/EpCAM(+) cells, stemness-related transcription factor expression, self-renewing ability and oxaliplatin resistance

**Figure 1:**
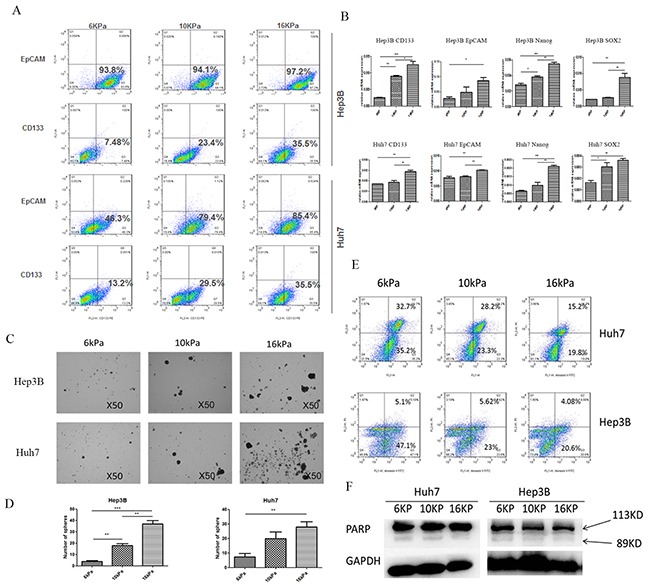
Higher matrix stiffness induces and enhances the stemness characteristics of HCC cells **A.** Increasing matrix stiffness enhances the proportion of cells with CD133(+)/ EpCAM(+) in two HCC cells; **B.** Higher stiffness remarkably upregulates the expression of CD133, EpCAM, and stemness-related transcription factor Nanog and SOX2 in two HCC cells. Error bars indicate SEM, ***, P value < 0.001; **, P value< 0.01; *, P value< 0.05. **C.** and **D.** Higher stiffness-induced HCC cells exhibit greater ability in morphology and quantity of spheres compared with the controls. Error bars indicate SEM, ***, P value < 0.001; **, P value< 0.01; *, P value< 0.05. **E.** Oxaliplatin resistance analysis shows that as the stiffness increases, the early apoptosis percentage and the late apoptosis percentage in the treated HCC cells are all descended. **F.** Low expression of PARP indicates the existence of higher stiffness-mediated oxaliplatin resistance in HCC cells.

Huh7 and Hep3B cells grown on COLI-coated polyacrylamide gel with different stiffness substrates (6, 10, and 16kPa) were harvested for the percentage analysis of cells with CD133(+) /EpCAM(+). The percentage of cells with EpCAM(+) in Hep3B cells in response to low, medium and high stiffness stimulation was 93.8%, 94.1% and 97.2% respectively, while that of CD133(+) was 7.48%, 23.4%, and 35.5%. In Huh7 cells, the percentage of cells with EpCAM(+) was 46.3%, 79.4%, 85.4%, and that of cells with CD133(+) was 13.2%, 29.5% and 35.5% (Figure 1A). These data demonstrate that increasing matrix stiffness may induce stem-like characteristics and enhance the amount of stem-like HCC cells. Moreover, higher stiffness also remarkably upregulated the expression of CD133, EpCAM, and stemness-related transcription factor Nanog and SOX2 in two HCC cells (Figure 1B). Except that, in sphere formation assay, higher stiffness-induced Huh7 and Hep3B cells also exhibited greater ability in morphology and quantity of spheres compared with the controls, thus suggesting that higher stiffness-induced HCC cells possess higher self-renewal ability (Figures 1C and 1D). As shown in oxaliplatin resistance analysis, the early apoptosis percentage in the treated Huh7 cells grown on 6, 10, and 16kPa stiffness substrate was 35.2%, 23.3%, and 19.8%, respectively, and late apoptosis was 32.72%, 28.2%, 15.2%. The early apoptosis percentage in Hep3B cells was 47.1%, 23%, 21.6%, and that in the late apoptosis was 5.1%, 5.62%, and 4.08%, indicating that higher stiffness stimulation enhances HCC cell resistance to oxaliplatin (Figure 1E). Additionally, low expression of apoptosis-associated protein poly ADP-ribose polymerase (PARP) which reflects caspase 3 activity in the treated HCC cells also confirmed the existence of higher stiffness-mediated oxaliplatin resistance in HCC cells (Figure 1F). All the above results illuminate that higher matrix stiffness may induce and enhance stemness characteristics of HCC cells.

**Figure 2:**
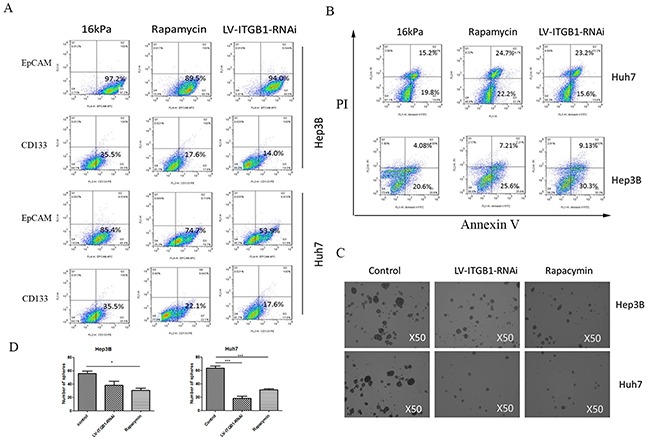
Integrin β1 knockdown and mTOR inhibitor reverse higher matrix stiffness-mediated HCC stemness characteristics **A.** The proportion of cells with CD133(+) or EpCAM(+) all decrease in the transfected HCC cells with LV-ITGB1-RNAi under higher stiffness stimulation. **B.** Oxaliplatin resistance analysis shows that higher stiffness-induced HCC cells with LV-ITGB1-RNAi present higher proportion of early and late apoptosis as compared with that of the controls. **C.** and **D.** The number of spheres derived from higher stiffness-induced HCC cells treated with LV-ITGB1-RNAi and Rapamycin also significantly diminish. Error bars indicate SEM, ***, P value < 0.001; **, P value< 0.01; *, P value< 0.05.

Integrin β1 as a leading different integrin subtype delivers matrix stiffness signal into HCC cells in our previous studies [14, 17]. Accordingly, we used HCC cells with lentivirus-mediated stable knockdown of integrin β1 to conversely validate that matrix stiffness-mediated effects on stemness properties indeed occur in HCC cells. Compared with that of the controls, the proportion of cells with CD133(+) and EpCAM(+) all decreased in the transfected Huh7 or Hep3B cells with LV-ITGB1-RNAi under higher stiffness stimulation (Figure 2A). In sphere formation assay, the number of spheres derived from higher stiffness-induced HCC cells with LV-ITGB1-RNAi also significantly diminished (Figures 2C and 2D). It suggests that the self-renewal ability of higher stiffness-induced HCC cells with LV-ITGB1-RNAi obviously decreased. Additionally, oxaliplatin resistance analysis showed that higher stiffness-induced Huh7 cells with knockdown of integrin β1 presented higher proportion of early and late apoptosis (22.2% and 24.7%) as compared with that of the controls (19.8% and 15.2%), and higher stiffness-induced Hep3B cells also had a similar trend in early and late apoptosis changes(7.21% and 25.6%; 4.08% and 20.6%) (Figure 2B). Taken together, we concluded that knockdown of integrin β1 can partially reverse higher stiffness-mediated effects on stemness characteristics in HCC cells.

### Higher matrix stiffness activates Akt/mTOR/SOX2 signaling pathway mediated by integrin β1 and contributes to HCC stemness

**Figure 3:**
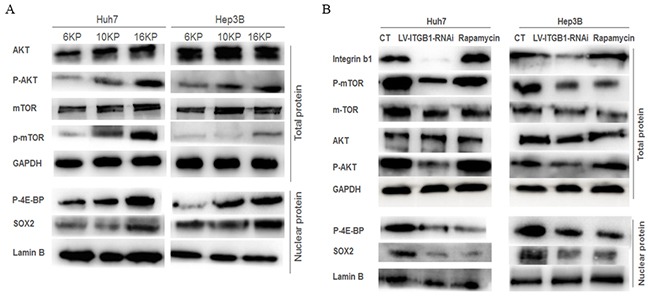
Integrin β1 and mTOR signaling pathway are involved in the regulation of matrix stiffness-mediated HCC stemness characteristics **A.** Increasing matrix stiffness upregulates the phosphorylation levels of Akt and mTOR, as well as the expressions of mTOR downstream molecules p-4E-BP and SOX2 in two HCC cells. **B.** Integrin β1 knockdown suppresses the phosphorylation level of Akt and mTOR, attenuates p-4E-BP and SOX2 expression in two HCC cells, while rapamycin, an mTOR-inhibitor, not only results in an obvious decrease of mTOR phosphorylation level and its downstream molecules expression, but also produces a slight increase in AKT phosphorylation level.

Subsequently, we explored the roles of mTOR signaling pathway in matrix-mediated effects on stemness properties in HCC cells. As the stiffness increased, the phosphorylation levels of Akt and mTOR were obviously upregulated, and the expression levels of mTOR downstream nuclear translocation factor, phosphorylated eukaryotic translation initiation factor 4E-binding protein (p-4E-BP), and stemness-related transcription factor SOX2 were also elevated (Figure 3A). This result certified that higher matrix stiffness stimulation may activate Akt/mTOR/SOX2 signaling pathway. Additionally, knockdown of integrin β1 suppressed the phosphorylation levels of Akt and mTOR, as well as the expressions of p-4E-BP and SOX2 in HCC cells grown on 16 kPa substrate stiffness (Figure 3B), thus suggesting that integrinβ1 mediates higher matrix stiffness-induced activation of mTOR pathway. These results are consistent with the changes of stemness phenotypes in HCC cells or HCC cells with LV-ITGB1-RNAi under different stiffness stimulation described above, implying that a link exists between integrin β1/Akt/mTOR/SOX2 signaling pathway and HCC stemness.

### mTOR inhibitor attenuates higher stiffness-induced stemness characteristics of HCC cells

Using mTOR-specific inhibitor rapamycin, we further validated the roles of mTOR signaling pathway in higher stiffness-induced stemness properties of HCC cells. Compared with that of the control, the phosphorylation level of mTOR and the expression of p-4E-BP and SOX2 all decreased in HCC cells treated with rapamycin (Figure 3B), and such results are similar to the changes of stemness characteristic under mTOR-specific inhibition (Figure 2). Intriguingly, a slight increase in AKT phosphorylation level, an upstream molecule of mTOR, was observed. This phenomenon is consistent with the results of other studies, and attributes to a loss of mTOR negative feedback [43, 44]. Meanwhile, the stemness properties of the treated HCC cells with mTOR inhibitor grown on higher stiffness substrate were all attenuated significantly, including the proportion of CD133(+)/ EpCAM(+) cells, self-renewing ability and oxaliplatin resistance (Figure 2B), further exhibiting an important regulating role of mTOR signaling pathway in matrix stiffness-mediated effects on stemness.

## DISCUSSION

CSCs are derived from normal stem cells that lose the ability to regulate proliferation or from somatic cells that acquire the capacities for self-renewal and tumor initiation after genetic lesions [45]. Lately, tumor cells dedifferentiate to possess stemness properties, which seem to be an alternative source of CSC [46, 47]. Different stemness markers may represent different cellular origins of CSCs, and CSCs with different origins maintain similar stemness characteristics including self-renewal, differentiation into multiple cell types, and other function properties like high clonogenic growth, high tumorigenic activity, and resistance to chemo/radiotherapy. Hypoxia, inflammation and liver stiffness are the most remarkable features of HCC microenvironment. Any of these stimuli can partially influence or determine the development and progression of HCC, and be associated with clinical outcomes closely [15, 16, 48–53]. Accumulating evidences illustrate that hypoxia and several inflammation cytokines regulate the stemness properties of HCC cells [54–56]. Hypoxia can induce stem cell markers such as octamer-binding transcription factor 4 (OCT4), NANOG, SOX2, kruppel-like factor 4 (KLF4), c-MYC, and microRNA-302 in 11 cancer cell lines including HCC cells through hypoxia-inducible factor (HIF) [57]. Low oxygen level after liver surgery may cause rapid loss of differentiation markers of tumor cells and increase the expression of CSC markers and the clone-forming capacity [58]. Interleukin-6 (IL-6)/STAT3 signaling upregulate the expression of CD133 in HCC cells and promote HCC progression [59]. Alteration of IL6 and Twist in hepatic CSCs significantly regulates microRNA expression and eventually influences chemoresistance and tumor spreading [60]. Additionally, HBx and HCV also induce cancer stem cell-like signatures and contribute to hepatocarcinogenesis [45]. Nevertheless, information is lacking on the relationship between matrix stiffness stimuli and HCC stem characteristic. Lately, OPN is found to promote a cancer stem cell-like phenotype in HCC cells via an integrin NF-κB-HIF-1α pathway [61]. Intriguingly, our previous data also suggested that higher matrix stiffness upregulated OPN expression in HCC cells [17], which implies a linkage between matrix stiffness and HCC stemness.

A number of signaling pathways have been reported to participate in stemness regulation. Other than activating β-catenin canonical pathway, Wnt also activates mTOR signaling pathway, a way concerning with the self-renewal of stem cells [62]. FAK/Akt, as the upstream of mTOR pathway and downstream of integrin, might play an important role in transducing extracellular mechanical signals and regulating HCC stemness [63, 64]. Cycling G1 upregulates SOX2 expression via Akt/mTOR signaling to promote liver tumor-initiating cells expansion [43]. Furthermore, mTOR signaling pathway is associated with stemness maintenance of embryonic stem cells (ESCs), and mTOR inhibition impairs pluripotency and hampers hESCs proliferation. Genome-wide microarray analysis reveals that the activation of mTOR signaling pathway downregulates the expression levels of growth and development-associated genes, thereby preserving the stemness of hESCs, and Nanog and SOX2 are involved in modulation of stemness preservation [65]. mTOR signaling pathway is activated in colorectal cancer stem cells, and correlated with the prognosis of colorectal cancer patients. Meanwhile, rapamycin, an mTOR inhibitor, decreases spheroid formation potential and ALDH expression, a marker of stemness [66]. These studies described above partially disclose some mechanisms of stemness maintenance, but little information is given to determine the mechanism underlying initiation by matrix stiffness signal.

In this study, we first discovered that HCC cells grown on higher stiffness substrate exhibited higher stemness characteristics as compared with those on lower stiffness substrate, indicating that higher matrix stiffness may induce or enhance the stemness characteristics in HCC cells. Subsequently, higher expressions in Akt and mTOR phosphorylation, as well as downstream molecules p-4E-BP and SOX2 revealed an important role of mTOR signaling pathway during matrix stiffness-mediated effects on HCC stemness. Integrin bridges the gap between extracellular physical signal and intracellular chemical signal [67]. The knockdown of integrin β1, identified as a leading integrin subtype to deliver stiffness signal into HCC cells [14, 17], attenuated higher stiffness-mediated stemness characteristics of HCC cells, and suppressed the phosphorylation levels of Akt and mTOR, as well as the expression levels of p-4E-BP and SOX2, suggesting that integrin β1 transmits higher stiffness signal into HCC cells, and drives the activation of mTOR pathway to direct stemness characteristic changes. mTOR inhibitor also significantly depressed the higher stiffness-induced stemness characteristics, and decreased the phosphorylation level of mTOR and the expression levels of p-4E-BP and SOX2, further confirming a specific regulation role of mTOR signaling pathway in matrix stiffness-mediated effects on HCC stemness. A different conclusion [12] was reported in another study that a soft matrix stiffness (1kPa) induced cellular dormancy, a stem cell phenotype, and enhanced clonogenic capacity following chemotherapy, while higher matrix stiffness (12 kPa) increased cisplatin resistance in HCC cells. Chemotherapy resistance and lower clonogenic capacity, two paradoxical properties for stem-like cancer cells, occur together in HCC cells grown on higher stiffness substrate. But in our study, three different stiffness gel with 6, 10 and 16 kPa were used to represent the stiffness level of a normal liver, fibrosis and cirrhosis, respectively [68], high matrix stiffness was found to enhance both high clonogenic capacity and oxaliplatin resistance. However, soft substrate gel with 1kPa as the control is far beyond the stiffness scope of liver disease, so it cannot mirror the matrix stiffness characteristic of diseases. Therefore, different stiffness scope may result in a distinct difference in the results of HCC stemness characteristics. Although a linkage between integrin/Akt/mTOR/SOX2 pathway and matrix stiffness-induced HCC stemness is clearly evident in the study, other co-regulation signal pathways are still not able to be excluded during this pathological process. Additionally, establishment of rat HCC model with tunable liver matrix stiffness backgrounds also deserve to be explored for better understanding matrix stiffness-induced HCC stemness in vivo.

**Figure 4:**
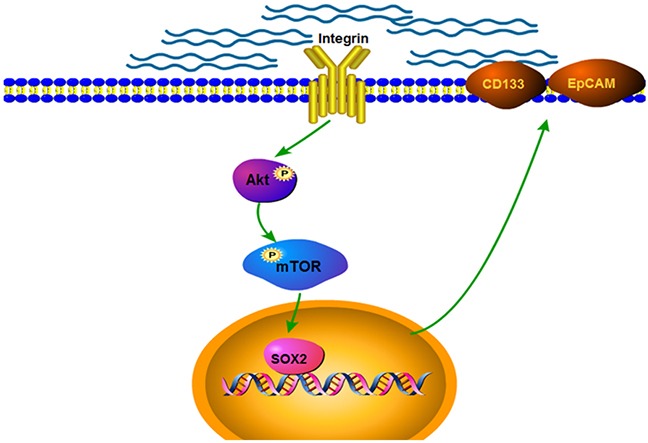
Schematic diagram of the proposed mechanism by which matrix stiffness drives integrin β1/Akt/mTOR/SOX2 signaling pathway to regulate the stemness characteristics of HCC

In summary, our data suggested that higher matrix stiffness, as an initiator, triggered and enhanced the stemness characteristics in HCC cells via activating integrin β1/Akt/mTOR /SOX2 signaling pathway (Figure 4). This study first unravels the obscurity between matrix stiffness and HCC stemness, and proposes a novel regulating pathway to direct HCC stemness.

## MATERIALS AND METHODS

### In vitro system of mechanically tunable COL1-coated polyacrylamide gel

An in vitro system of mechanically tunable COL1-coated polyacrylamide gel was established as previously described [13, 16]. Polyacrylamide gels with different mechanical stiffness levels were prepared by mixing 10% acrylamide(Sigma USA) and 0.01%–0.5% bis-acrylamide (Sigma USA) in a HEPES-buffered solution (pH 8) supplemented with 10% ammonium persulfate (1/100 volume) and TEMED (1/100 volume). The formed gels were further crosslinked and coated with 0.1 mg/ml of COL-1 solution (BD) suitable for cell culture.

### HCC cells and cell culture

Huh7 cells (ATCC, USA) were cultured in Dulbecco's modified Eagle's medium (Gibco, USA) supplemented with 10% fetal bovine serum (FBS; Biowest, South America Origin) and 1% penicillin/streptomycin (Gibco, USA). Hep3B cells (ATCC, USA) were cultured in minimum essential medium (Gibco, USA) supplemented with 10% FBS and 1% penicillin/streptomycin. Approximately 3×10^5^ HCC cells in 0.3 ml of medium were seeded onto a thin layer of COL1-coated polyacrylamide gel with tunable stiffness for 2 h at room temperature. Subsequently, 12 ml of culture medium was added to the dish, and the cells were further incubated at 37°C for 24 or 48h.

### Western blot and reverse transcription-PCR

Supplementary Materials and Methods provide the detailed procedures of Western blot and RT-PCR.

### Stable knockdown expression of integrin β1 in HCC with lentiviral vectors

Small interfering RNAs (siRNAs) targeting the human integrin β1 gene were designed by the Shanghai GeneChem, Co., Ltd., China. The optimal sequence of siRNA against human integrin β1 (5b 2-CCTCCAGATGACATAGAAA-3b 2) was then cloned into the plasmid GV112. Lentivirus preparations were produced by Shanghai GeneChem, Co. Ltd, China. The resulting siRNA human integrin β1 sequence was confirmed by PCR and sequencing analysis. Different siRNAs were screened by cotransfection of a human integrin β1 cDNA plasmid into HEK293T cells with Lipofectamine 2000 (Invitrogen Corporation, Carlsbad, CA, USA). The viral supernatant was harvested 48 h after transfection, and the viral titer was determined. The viral supernatant was added into the target HCC cells (at multiplicity of infection=10) with ENi.S and 5 μg/ml of polybrene to obtain stably-transfected HCC cells with integrin β1 knockdown.

### Rapamycin intervention experiment

Rapamycin is a classic mTOR pathway activation inhibitor. Huh7 and Hep3B cells were cultured on different stiffness substrates for 24 h. About 100nM of Rapamycin (Selleck, China) was added into medium and culture was continued for another 24 h. Cells were harvested for further experiments.

### Flow cytometry (FCM) analysis

The apoptosis of HCC cells grown on different stiffness substrates was detected by Annexin-V-FLUOS Staining Kit (Roche, Germany). About 10^6^ of HCC cells were washed with 1×PBS and centrifuged at 2000 rpm for 5 min. Subsequently, the cell pellets were resuspended in 100 ml of Annexin-V-FLUOS labeling solution and incubated for 10 min at room temperature. Cell apoptosis was analyzed using FCM with a 488, 515, and >600 nm of excitation wavelength bandpass filter for fluorescein detection and filter for PI detection, respectively.

CD133/1(AC133)-PE and CD326(EpCAM)-APC antibody (Miltenyi, Germany) were used to detect the proportion of HCC cells with stemness marker CD133(+)/EpCAM(+). Approximately 10^6^ of Huh7 and Hep3B cells cultured on different stiffness substrates were collected. The cells were washed with 1× PBS, and resuspended with 80μl buffer, added in 20μl FcR Blocking Reagent and 10μl CD133 or CD326 antibody, respectively. The cells were incubated for 10 min at 4°C and washed with PBS twice. FCM was used for analysis using an excitation wavelength in 488nm (CD133) and 633nm (EpCAM), as well as a 515 nm bandpass filter for fluorescein detection.

### Sphere formation analysis

HCC cells were trypsinized into single cells and resuspended in serum-free DMEM/F12 medium (Gibco, USA). Subsquently, they were transferred into a six-well plate with low attachment (Corning, USA) according to a density of 800 cells/well. EGF (4 ng/ml) and β-FGF (2 ng/ml) were added every second day. The cells were photographed using phase contrast microscope after 7 days of culture

### Statistical analysis

Statistical analysis of values for comparison among three groups was performed using One-way ANOVA. Data were expressed as mean ± SD, and p < 0.05 was considered statistically significant.

## SUPPLEMENTARY MATERIALS AND METHODS


