# Supplementary material for: Matrix stiffness-mediated effects on stemness characteristics occurring in HCC cells
Source: Oncotarget. 2016 Mar 31;7(22):32221–31. doi: 10.18632/oncotarget.8515 (PMC5078009; doi:10.18632/oncotarget.8515)
Supplement: Supplementary file 1 [file oncotarget-07-32221-s001.pdf]

SUPPLEMENTARY DATA

MATERIALS AND METHODS

Western blot

After 48 h culture on COL1-coated polyacrylamide gels with tunable stiffness, HCC cells were collected using a cell scraper from the gel. The total proteins of HCC cells were extracted in the mixed lysis buffer containing RIPA buffer (Beyotime, China), 1 mM phenylmethanesulfonyl fluoride (Beyotime, China), and 10% PhosSTOP (Roche, Switzerland). Approximately 50 mg of total protein was loaded and separated by 10% SDS-PAGE and then transferred onto a polyvinylidene difluoride membrane (Millipore, USA). Subsequently, the membrane was blocked with 5% fat-free milk in TBS/Tween for 1 h at room temperature and reacted overnight at 4°C with the following diluted primary antibodies: integrin β1(1:1000, Cell Signal Technology, Danvers, MA), AKT (1:1000, Cell Signal Technology, Danvers, MA), p-AKT(1:1000, Proteintech, Wuhan, China), mTOR(1:1000, Cell Signal Technology, Danvers, MA), p-mTOR (1:1000, Cell Signal Technology, Danvers, MA), p-4E-BP (1:1000, Cell Signal Technology, Danvers, MA), SOX2 (1:1000, Cell Signal Technology, Danvers, MA), GAPDH (1:1000, Cell Signal Technology, Danvers, MA), and lamin B(1:1000, Cell Signal Technology, Danvers, MA). The membrane was further incubated with HRP-conjugated secondary antibody (1:1000, Dingguo Bio Beijing, China) for 1 h at room temperature. Finally, the target band was visualized using an electrochemiluminescence kit (Thermo, USA).

RNA extraction and RT-PCR assay

Total RNA was extracted from cultured cells with TRIzol Reagent (Invitrogen). The quality of RNA was examined by A260 absorption. For mRNA detection, about 2 μg of total RNA was used for complementary DNA synthesis with a RevertAid/First Strand cDNA Synthesis Kit (Thermo Fisher Scientific, Inc.). RT-PCR was performed in triplicate using Platinum SYBR Green qPCR SuperMix-UDG (Invitrogen, Inc.). The primers for the genes were synthesized by Sangon Biotech Co., Ltd., as follows:

CD133: 5′- AGTCGGAAACTGGCAGATAGC-3′(forward) and 5′- GGTAGTGTTGTACTGGGCCAAT-3′(reverse).

EpCAM: 5′-AATCGTCAATGCCAGTGTACTT-3′ (forward) and 5′- TCTCATCGCAGTCAGGATCATAA-3′ (reverse).

Nanog: 5′- TTTGTGGGCCTGAAGAAAACT-3′ (forward) and 5′- AGGGCTGTCCTGAATAAGCAG-3′ (reverse).

SOX2: 5′- GCCGAGTGGAAACTTTTGTCG-3′ (forward) and 5′- GGCAGCGTGTACTTATCCTTCT-3′ (reverse).

GAPDH: 5′-TGTGGGCATCAATGGATTTGG-3′ (forward) and 5′- ACACCATGTATTCCGGGTCAAT-3′ (reverse).
